# Hybrid Polymer–Inorganic Salt Hydrate Materials: Applications for Heat Storage and Beyond

**DOI:** 10.1021/acsami.6c07502

**Published:** 2026-06-01

**Authors:** Kartik Kumar Rajagopalan, S. M. Ashik Abedin, Peiran Wei, Svetlana A. Sukhishvili

**Affiliations:** Department of Materials Science and Engineering, 14736 Texas A&M University, College Station, Texas 77843, United States

**Keywords:** inorganic salt hydrates, salogels, thermal energy storage, phase change materials, supercooling

## Abstract

Polymer–salt hydrate hybrid materials called salogels combine the key properties of polymers, such as flexibility, with the high heat of fusion and inflammability and high salt content of the inorganic salts. These features make them promising materials for thermal energy storage and conversion, personal thermal management, anti-icing, and shape-morphing applications. However, further development is constrained by supercooling issues, poor control of mechanical properties, and limited reprocessability and scalability. This perspective briefly reviews recent applications of salogels, highlights the importance of advancing understanding of the specific mechanisms of interactions between polymer networks and inorganic salt ions to overcome these challenges, and outlines future directions in the development of polymer–salt hydrate hybrid materials. We suggest that by leveraging understanding of polymer–salt hydrate interactions, future research can focus on the rational development of processable polymer networks with controlled temperature-responsive gelation for specific salt hydrate or salt hydrate eutectics and the ability to suppress supercooling for robust thermal and mechanical performance. The perspective further identifies the importance of recruiting ML and additive manufacturing techniques for the accelerated development of polymer–salt hydrate hybrid materials of diverse chemistry and shapes for broader applications, which combine thermal energy storage with applications exploiting ionic mobility, such as electrochemical conversion, supercapacitors, and polymer electrolytes.

## Graphical abstract

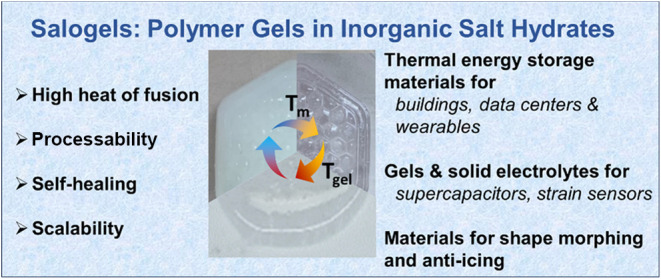

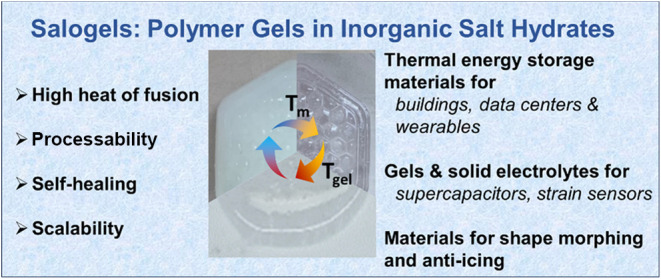

## 1 Introduction

Limited fossil fuel reserves and the intermittent nature of renewable (e.g., solar and wind) energy sources have stimulated the development of materials and technologies for energy conversion and storage.^1,2^ One promising thermal energy storage (TES) technology is based on phase change materials (PCMs), which are capable of reversibly storing a large amount of thermal energy in the form of latent heat during solid–liquid phase transitions over a small temperature range.^1,3,4^ PCMs can be classified as organic, inorganic, and eutectics.^1,5^ Compared to organic PCMs, inorganic PCMs such as inorganic salt hydrates (ISHs), AB·*n*H_2_O (A-cation, B-anion), are highly valued due to high volumetric latent heat of fusion (up to 450 MJ/m^3^ compared to 200 MJ/m^3^ for paraffin wax) and nonflammability.^5,6^ These features are critical for applications of these materials for cooling buildings when directly incorporated within building walls or integrated within the AC systems, electronic components, and batteries^7^ or for providing comfort to the human body when integrated within textiles, wearable devices, and biomedical patches.^3,8^ An additional benefit is the low cost and easy availability of several ISHs, such as CaCl_2_·6H_2_O, which is a byproduct of soda ash manufacturing, making them desirable for the development of scalable TES materials.^9,10^ However, the widespread use of ISH PCMs has been impeded by their low viscosity in the molten state, leading to leakage, phase segregation (for incongruently melting salt hydrates), and a high degree of supercooling.^2,5,8,11^ In addition, ISHs are more rigid and brittle in the solid state due to their higher density as compared to their softer, more flexible organic counterparts.^5^ This rigidity presents an additional challenge for application in wearable devices, where deformability of TES materials is critical. Therefore, strategies for shape stabilization of ISHs that also provide flexibility are needed.

The approaches developed for transforming ISHs into robust TES materials mostly focused on shape stabilization and used strategies similar to those developed for much more studied organic PCMs, such as paraffins.^8,12–15^ These approaches include entrapment within porous ceramic, metallic, or polymer support matrices, core–shell encapsulation, and more recently developed shape stabilization by gelled polymers.^2,8,11,16^ Among these approaches, the use of ISH-insoluble porous support matrices and encapsulation shell materials dramatically limits achievable PCM loads (which are typically below 70%), thus reducing the materials’ thermal energy storage density.^13,17–26^ In addition, encapsulation of salt hydrates has proven to be challenging due to their intrinsic properties, such as high fluidity in the liquid state, phase separation of incongruently melting salt hydrates within the core of the capsule, and significant (up to 10%) volume expansion upon melting^20^ and suffers from complex procedures, presenting an additional barrier for a scale-up. Finally, the above techniques do not offer a facile and efficient route for processing and reprocessing of TES materials—a feature that is critical for applications. Dissolving polymers in liquid ISHs (i.e., the use of polymer thickeners) has been long used to increase their viscosity, but this strategy failed to yield shape-stable ISH materials.^27–30^ Entrapping salt hydrates within permanently crosslinked polymer networks was then pursued as a means to provide PCM shape stability at a minimal content of a polymer component, leading to a high retention of the pristine ISH heat of fusion (up to 90%).^31–52^ The technique is easy to implement by dissolving the monomer or polymer in the salt hydrate followed by crosslinking either through polymerization or the use of crosslinkers.^31–52^ However, the use of permanent polymers networks, similar to permanent scaffolds, reduces TES capacity due to low PCM loadings and does not allow on-demand tunability of mechanical properties and reprocessing of TES materials.

Rendering polymer networks dynamic via introducing non-covalent polymer–polymer interactions (hydrogen bonding, dynamic covalent bonding, and/or entanglements) has recently emerged as a unique means for transforming ISHs into processable TES materials.^9,53–55^ In addition to processability, next-generation polymer–ISH materials should have a unique combination of thermomechanical properties to become suitable for energy applications. They should remain shape-stable and self-healable in the proximity of ISH melting temperatures and yet become easily processable on demand for integration within or removal from TES devices. They should be able to shape-morph and manifest mechanical properties that allow them to conform to heat exchanger surfaces or a human body for maximized heat transfer efficiency and comfort and should be able to do so not only in the liquid but also in the solid state of a salt hydrate. Moreover, polymer–salt hydrate PCMs should provide intrinsic or extrinsic mechanisms to suppress supercooling for a maximized heat storage capacity. Finally, to broaden applications, inorganic salt-based materials should target areas beyond latent heat-based energy storage, such as solar energy and electrochemical energy conversion, and should be easily integrated with other materials (photothermal converters, nucleation enhancers, etc.). Notably, the above characteristics should be achieved while minimizing the content of the shape-stabilizing matrix, nucleation-controlling agents, etc., for the maximized energy storage capacity.

Prior reviews provide excellent overviews of the applications and strategies for shape stabilization of both organic and inorganic PCMs, focusing on thermal and energy storage properties rather than the molecular design of TES materials.^1,8,11–13^ Recent advances in using polymer gels for shape stabilization of organic and inorganic PCMs, including material design, have also been reviewed.^2,56^ However, the state-of-the-art and future developments of the novel type of gels in the high-salt environment of salt hydrates have not yet been discussed. This perspective identifies the current knowledge gap in correlating molecular interactions in the polymer-stabilized salt hydrates with the overall material performance while also exploring the untapped potential of polymer-stabilized salt hydrates in fields beyond traditional energy storage. One of the aims of this perspective is to highlight the critical issues and needs in designing polymer networks for salt hydrates in the unique extreme salinity nature of ISHs,^3,57^ focusing on developing high-heat of fusion, mechanically tunable, reprocessable polymer gels in salt hydrates for diverse energy applications. We name these gels “salogels” ^3,53,58^ to underscore the distinct nature of liquid ISHs as solvents (extremely high, 9–18 mol/kg, concentration of salt ions, leading to the unsaturated first hydration shells^3,59^ ) and differentiate salogels from hydrogels in which water is abundant.^2,56^ This terminology is consistent with the terminology used for polymer gelation in other solvents, such as ionic liquids (ionogels)^60,61^ or organic solvents (organogels).^62–64^ The term “salogel” also highlights the critical roles of the salt ions in polymer solubility that can lead to highly selective salting-in and salting-out behavior of polymer chains in ISH solvents, such as facile solubilization of cellulose, which is difficult to dissolve in common organic solvents and water,^65,66^ and insolubility of many common polyelectrolytes which are highly soluble in water and aqueous solutions with low and moderate salt concentrations.

In this perspective, we discuss the recent advances in designing salogels by using dynamic, noncovalent interactions and dual networks to endow self-healing and the use of environmental triggers to achieve reprocessable TES materials and identify future research and material design needs. We then highlight the unique opportunities in applications of these materials in leveraging combined properties of the ISH and the dynamic nature of polymer networks to develop multifunctional materials. The applications for these materials range from traditional thermal management in buildings and wearable devices for personal thermal management to newer applications such as energy conversion devices, supercapacitors, gel electrolytes, anti-icing coatings, and smart windows (Figure 1). We also identify the potential of the polymer network molecular design and ISH confinement in suppressing salt hydrate supercooling as a critical bottleneck to widespread applications. Finally, we outline strategies for using the polymer matrix to endow mechanical compliance both in the ISH liquid and solid state required for seamless integration to interfaces for applications of salogels in personal thermal management, strain sensors, artificial skin, smart windows, and shape-morphing materials.^67–69^

**Figure 1:**
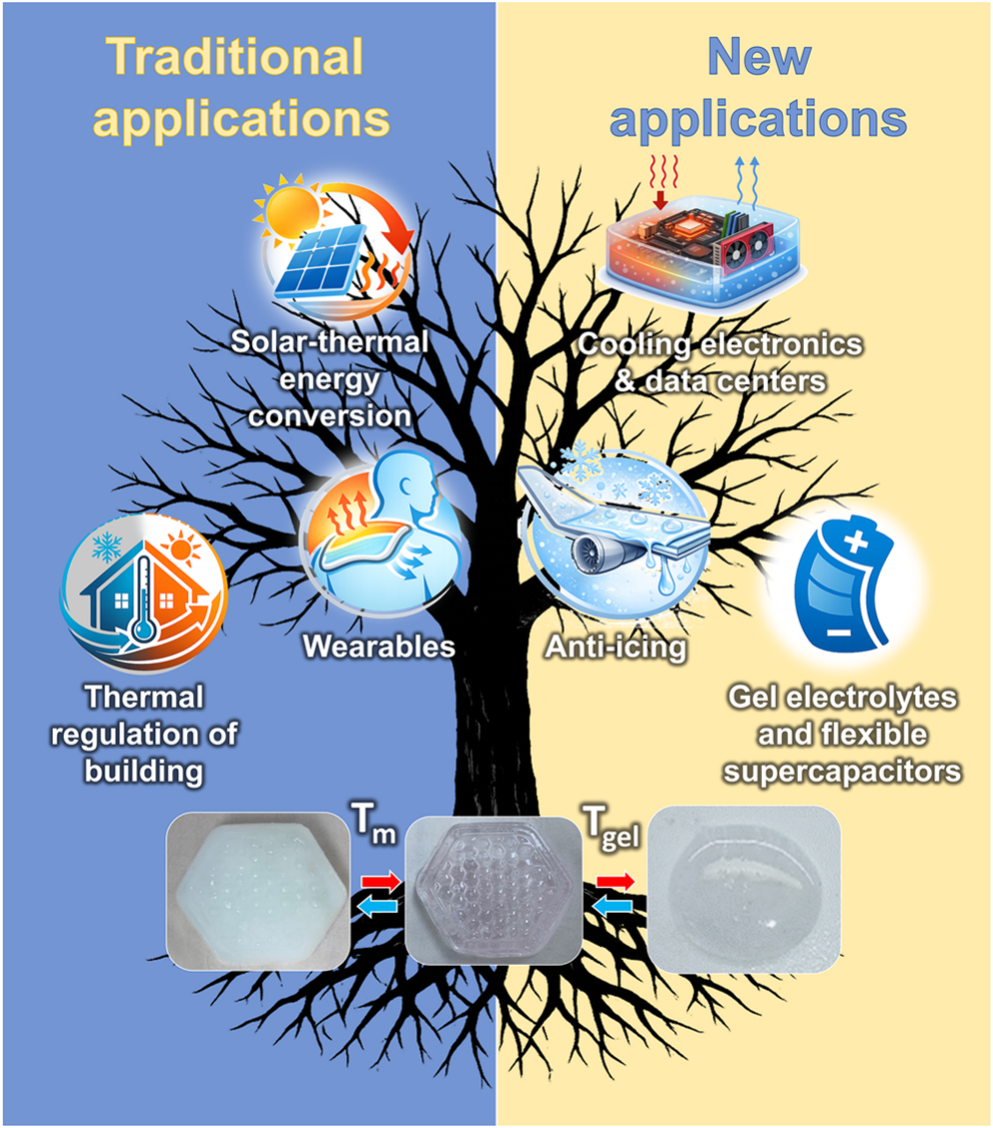
Schematic showing traditional and emerging applications of salogels. Images of salogels are reproduced with permission from ref 54. Copyright 2022 Royal Society of Chemistry.

## 2 Salogels: Polymer–Salt Hydrate Hybrid Materials

### 2.1 Salt Hydrates as Unique Polymer Solvents

Shape stabilization of molten ISHs by trapping within permanent polymer networks typically involves dissolving the monomer or polymer in the salt hydrate followed by thermal or photoinitiation of the polymerization reaction or addition of a cross-linker to induce gelation.^9,31–55,70^ However, the wide use of these strategies is hampered by the lack of an understanding of fundamental polymer dissolution and network formation in these solvents. The high ionic and low water concentrations lead to incomplete saturation of the hydration shell of the ions and a complex ionic environment with extensive formation of contact and solvent-shared ion pairs^3,57,71^ and weakened hydrogen bonding among the water molecules.^3^ ISHs can also be considered as dynamically evolving ion–water networks,^72^ where ion transport is strongly coupled to the ion–water structure, ion association, and hydrogen bonding.^71,73,74^ The balance of ion–ion and ion–water interactions also governs ionic conductivity, which follows a hopping mechanism as compared to vehicular diffusion in aqueous salt solutions.^71,72^ The ion transport provides an opportunity to transform ISHs from purely TES materials into multifunctional platforms for electrothermal energy conversion and storage, gel electrolytes, and supercapacitors. Compared to conventional ceramic, polymer, and hydrogel electrolytes, salogel electrolytes mitigate the inherent trade-offs between mechanical strength and ionic conductivity, offering a balanced combination of high modulus, efficient ion transport, wider operating voltage, and enhanced thermal tolerance.^39^

In the extreme salinity environment of salt hydrates, polymers remain dehydrated and their solubility and gelation become dramatically dependent on the changes in water content and chemistry of cations or anions due to the Hofmeister effect.^75^ However, very few studies exploring the fundamentals of polymer–ISH interactions exist. Our group previously used spectroscopic techniques, attenuated total reflectance-Fourier transform infrared spectroscopy (ATR-FTIR),^3,58^ and ^7^Li nuclear magnetic resonance (NMR)^76^ to demonstrate the effect of the ion type and water content on gelation of polar, neutral polymers (such as PVA) in salt hydrates. Gelation was shown to be facilitated by the dehydration of polymer chains accompanied by the coordination of the cation with the functional group on the polymer backbone. A competition between the ions and polymer for the scarce water results in the formation of polymer–polymer hydrogen bonds and polymer–ion interactions that support gelation even in the absence of a cross-linker.^3,54^ In contrast, gelation of PVA in water to form hydrogels requires the addition of a crosslinker or additional processing such as freeze-thawing.^77,78^ In further work, ATR-FTIR was used to demonstrate an extreme Hofmeister effect and ion type on the gel strength of a salogel in a chloride and a nitrate salt hydrate.^9^ This work underscores the importance of understanding polymer–ISH interactions and how this can drive the rational design of polymer networks for their shape stabilization. However, the gelation driven by polymer–ISH interactions alone is not sufficient for mechanical strength improvement, and crosslinking (covalent or dynamic) is needed for robust mechanical stability.

Theoretical and computational studies are also critical for fundamental understanding of the behavior of polymer chains in salt hydrate solvents. Recently, Yin et al. used density functional theory (DFT) calculations to compare the solubility of PVA, polyacrylamide (PAAm), and poly­(acrylamide-*co*-hydroxyethyl methacrylate) (p­(AAm-*co*-HEMA)) in water and LNH and showed that the ISH is a better solvent for these polymers due to favorable interactions between the ions and polar functional groups on the polymer chain resulting in an overall lower energy state.^32,57^ Wei et al. used DFT calculations to demonstrate improved solubility for gels of PAAm and poly­(3-dimethyl­(methacryloxyethyl)­ammonium propanesulfonate) in sodium acetate trihydrate (SAT) compared to water driven by polymer–ion interactions.^39^ Such comparison studies using computational tools for other combinations of salt hydrates and polymers could drive the rational design of these gels in the future, especially in eutectic salt hydrates where the presence of multiple types of cations and anions adds another level of complexity in understanding the polymer solvation and network formation. Moreover, combining computational tools with machine learning (ML) and high-throughput (HT) experimentation could aid the progress in understanding polymer physics of polymers that have thus far not been explored in this field.

### 2.2 Polymers in Salt Hydrates: Covalent vs Dynamic Networks

Shape-stabilizing matrices in salogels are typically constructed using hydrophilic polar polymers such as poly­(vinyl alcohol) (PVA), poly­(acrylamide) (PAAm), and poly­(acrylic acid) (PAA) and some charged polymers such as sodium salt of poly­(acrylic acid) (PAAS) or alginates. For example, several studies report covalently crosslinked gels (Figure 2A) of these polymers for shape stabilization of various salt hydrates such as LiNO_3_·3H_2_O (LNH),^33^ Na_2_HPO_4_·12H_2_O (DPDH),^51,52,79^ Na_2_SO_4_·10H_2_O (SSD),^44,46,80,81^ and CH_3_COONa·3H_2_O (SAT)^34,38,39,41,69,82–88^ and some eutectic salt hydrates.^31,47–49,89–91^ Initially, these efforts were focused on developing covalently cross-linked single polymer networks which provided shape stability and mechanical strength (10–1000 kPa). However, the heat of fusion of these salogels was relatively low (50–80%) (Figure 2B and Table 1) and the gels lacked self-healing capability.^31,33,36,42,45–47,52,57^ To introduce self-healing capability, double-network approaches combining ionic crosslinking or hydrogen bonding with covalent crosslinking were employed.^32,35,37,40,43,45,50,51,70,79,84,85,92^ In these gels, the covalent crosslinks provide elasticity and strength to the network, essential for shape stabilization, while the weaker ionic or hydrogen bonds endow flexibility and self-healing capability to the network essential for wearable device applications. Typically, the first network was constructed using PAAm or PAAS covalently crosslinked with bis­(acrylamide) and the second network was either formed by polymers capable of forming ionic crosslinks^35,43,45,50,70,92^ or hydrogen bonds.^36,37,40,79,84,85^ The presence of the secondary networks served dual purposes of introducing self-healing (with a healing efficiency of 75–88% for hydrogen bonding and 40–60% for ionic cross-linking)^37,40,43,45,84,92^ and controlling mechanical properties. However, the addition of the second polymer network largely reduces the heat of fusion (Figure 2B), compromising the material’s thermal performance, and the presence of covalent crosslinks impedes the processability of these materials. Specifically, the resultant salogels cannot be reshaped and repurposed and are difficult to 3D print and/or to fill in or remove from the casings at the end of the life.

**Figure 2:**
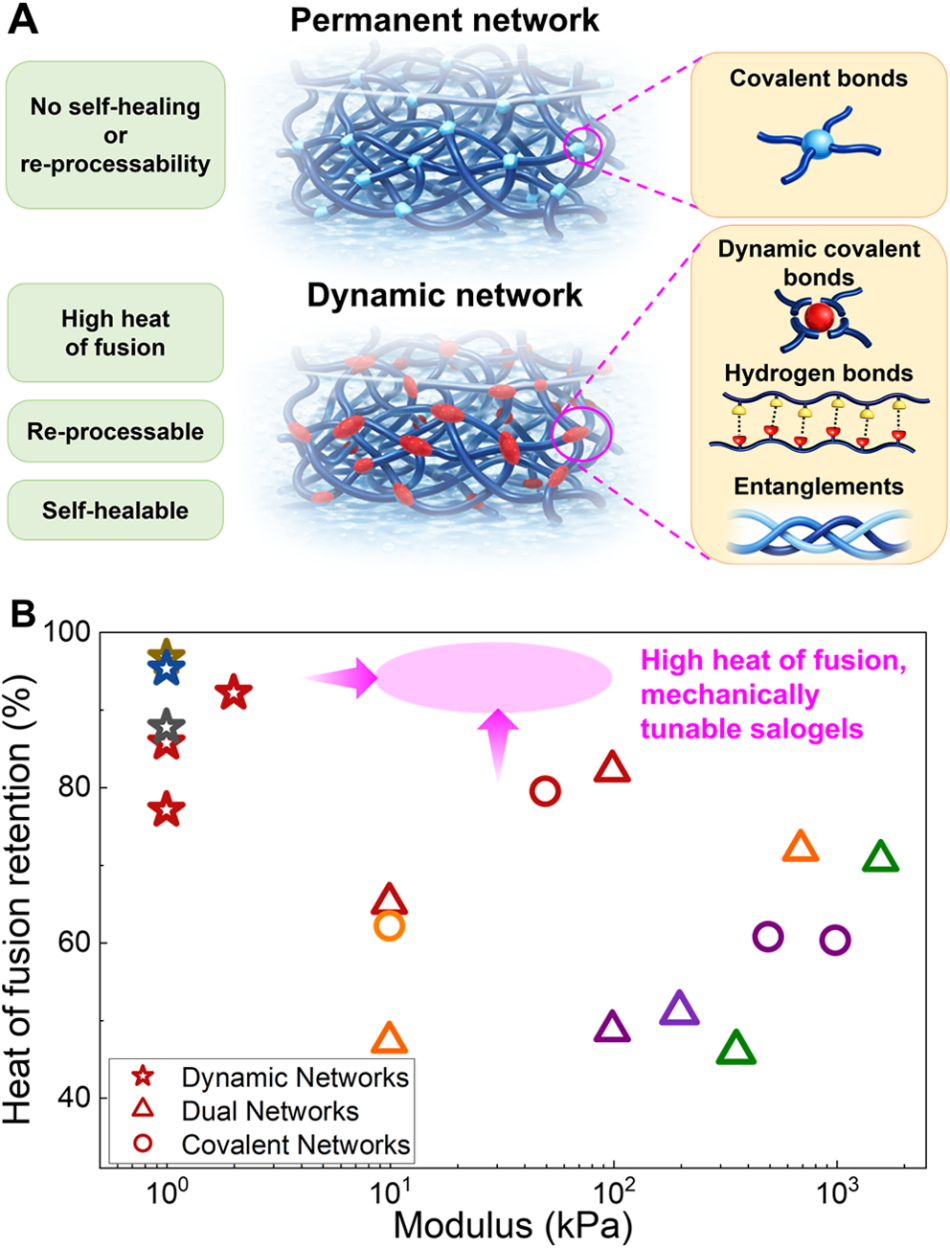
(A) Schematic showing covalent and dynamic crosslinking strategies in salogels. (B) Comparison of % heat of fusion retention in the salogel (comparison to a neat ISH) and modulus of the gel. The arrows and blue box indicate the desired combination of TES capacity and modulus for next-generation salogels, respectively. Color coding for data points: red: LNH, gray: MgNH, blue: CCH, green: SAT, orange: SSD, purple: DHPD, and dark yellow: CNH. Data taken from refs 9,31–33,36,43,45,51–55,57,58,79,80,85 .

**Table 1:** Summary of the Properties of the Covalent, Dual, and Dynamic Network Salogels

Salogel type	ISH	heat of fusion retention (%)	elastic modulus (kPa)	melting temperature (°C)	crystallization temperature (°C)	features	refs
covalent network	LNH	82	100	29	–4	Pros: shape stability and mechanical strength	^57^
	LNH	65	10	29	–26		^33^
	SAT	70.5	1600	58	56.8		^36^
	SSD	62	10	32	-		^80^
	DHPD	48.6	100	36	-	Cons: low heat of fusion and lacks self-healing and reprocessability	^52^
	DHPD	60.2	1000	36	-		^31^
	DHPD	60.7	500	36	34.6		^51^
Dual network	LNH	79.5	50	29	-	Pros: shape stability and self-healing	^32^
	SSD	47	10	32	-		^43^
	SSD	71.9	700	32	-		^45^
	DHPD	50.9	500	36	-	Cons: low heat of fusion and lack of reprocessability	^79^
Dynamic network	LNH	77	1	29	–6	Pros: high heat of fusion, shape stability, self-healing, and reprocessability	^58^
	LNH	85.7	1	29	–20		^53^
	LNH	92.1	2	29	–2		^55^
	MgNH	87.7	1	89	78		^55^
	CNH	96.7	1	43	-	Cons: low mechanical strength	^54^
	CCH	95.2	1	29	0		^9^

One approach to overcome these drawbacks involves the use of dynamic covalent crosslinks in polymer gel networks to shape-stabilize the salt hydrates (Figure 2A,B). Dynamic covalent crosslinks can support networks for trapping ISH PCMs and also render the materials reprocessable, reshapable, and self-healable. These properties are endowed by the ability of the cross-links to undergo bond exchange or dissociation in response to different stimuli such as temperature, pH, or radiation. While the use of dynamic covalent crosslinking has been demonstrated with organic PCMs,^93–101^ this approach has been reported only recently for ISH PCMs, i.e., salogels. Our group developed thermo-reversible salogels using dynamic non-covalent (hydrogen bonding and entanglements)^9,53^ and dynamic covalent crosslinking (boronate ester and Diels–Alder (DA))^9,54,55^ approaches for shape stabilization of nitrate and chloride salt hydrates. These thermo-reversible salogels showed self-healing efficiency up to 95%, effectively shape-stabilized salt hydrates in their molten state, but at a temperature well above the melting point of the PCM, called the gel-to-sol transition temperature (*T*_gel_), could be deconstructed on demand, converting the gel to a high-viscosity sol. Below *T*_gel_, the polymer network provided shape stabilization of the salt hydrate, whereas above *T*_gel_, the viscous sol could be processed into different shapes using molding or 3D printing. The *T*_gel_ can be controlled by the crosslinking chemistry and in the case of combined hydrogen bonding and boronate ester cross-linking is tunable between room temperature and 75 °C.^9,53,54^ Thus, a temperature gap of more than 30 °C (*T*_gel_–*T*_m_ > 30) could be achieved for boronate ester salogels, allowing a wide temperature window of operation for the salogel. On the other hand, the stronger dynamic covalent cross-links, such as those based on the Diels–Alder (DA) furan-maleimide click reaction, demonstrated the capability to shape-stabilize salt hydrates over a wider temperature window (*T*_gel_–*T*_m_) ranging from ∼60 °C for a high-melting PCM (*T*_m, MgNH_ 89 °C) to 120 °C for a ambient melting PCM (*T*_m, LNH_ 29 °C).^55^ In addition, the “click” nature of DA crosslinks allows faster gelation, and DA salogels remained resistant to creep at high temperatures while still being thermoresponsive and self-healable.

The on-demand reversibility of dynamic salogels offers opportunities for processing beyond traditional molding, such as in direct ink writing. For example, dynamic salogels can be extruded through a nozzle in the sol state above the *T*_gel_ as a printing ink, while cooling below *T*_gel_ will allow gelation and shape retention after printing. While 3D printing has been explored significantly with organic PCMs and has been used to create complex geometries for heat exchange modules or simply to deposit PCM gels onto fabrics for wearable device applications,^102^ additive manufacturing of salogels remains unexplored. While molding has been extensively used to prepare both covalent and dynamically crosslinked salogels into TES materials of different shapes and sizes, future research can focus on the new processing techniques for dynamic salogels.

In addition, Figure 2B illustrates the material design challenge for achieving salogels that simultaneously demonstrate high thermal energy storage capacity and can be designed to exhibit an elastic modulus tunable within a broad range for specific applications. In particular, compared to covalent and dual polymer networks, the single dynamic covalent networks developed so far show high retention of heat of fusion (80–95%) due to the low polymer content (<10 wt %) to ensure that the TES capacity of the gel is maximized but have lower mechanical strength. Thus, future research can also focus on exploring the role of polymer architecture in improving the control over mechanical properties of the salogels while simultaneously retaining a high salt hydrate content. Approaches to improving mechanical strength at low polymer concentration could involve the use of branched or star polymer architectures, which can increase crosslinking density and improve network homogeneity by reducing network defects from random distribution of crosslinking points.^103,104^

The other concern for applications of polymer-stabilized ISHs in personal management devices is flexibility in both liquid and crystalline states. While salogels remain flexible in the molten state of the salt hydrate, they can become brittle after crystallization due to a 3–6 orders of magnitude increase in the modulus of solid salt hydrates, leading to brittle fracture and poor material compliance. The requirement of flexibility of materials used as wearables is universal, affecting the design of wearable patches for health monitoring that are made of a broad spectrum of materials.^105,106^ In the case of salogels, Yin et al. have recently demonstrated a strategy to achieve flexibility even in the salogel crystalline state using a combination of double network and solvent modification approaches. The double network consisting of PVA and in situ polymerized p­(AAm-*co*-HEMA) in combination with a small amount of excess water (5–10%) showed remarkable flexibility even in the crystalline state of the PCM.^32^ However, this flexibility was achieved at the cost of thermal energy storage capacity (150–180 J/g compared to 280 J/g for neat LNH) of the salogel due to the use of excess water and high polymer concentration (∼25 wt %).

### 2.3 Polymer-Driven Supercooling Reduction in Salt Hydrates

An important parameter for retention of the high TES capacity of salogels is controlling the crystallization of the ISH trapped in the polymer networks (schematic in Figure 3). Crystallization requires the formation of a critical nucleus whose free energy reflects a competition between bulk driving force and interfacial energy, producing an activation barrier that must be overcome before stable growth can proceed.^107^ When this barrier remains high, the liquid phase can persist far below its equilibrium melting temperature, leading to pronounced supercooling. Neat ISH PCMs can show supercooling anywhere from 10 to >50 °C, impeding their widespread applications in energy storage (Figure 3).^9,55,108^ Supercooling of ISHs manifests itself in substantial variations in the induction time and the crystallization temperature under identical macroscopic conditions, which are caused by the stochastic formation of critical nuclei.^107^ The uncontrolled supercooling renders the discharge temperature and timing unreliable, making suppression of supercooling an essential focus in TES applications.^109,110^ Traditionally, inorganic particles, such as dispersed nucleating agents or crystallization seeds, have been added to ISHs to promote heterogeneous nucleation and reduce supercooling; however, such particles may need to be incorporated in amounts varying from 0.5 to 10% ^19,35,37,42,44,45,51,83,84,89^ to be effective and can lead to practical limitations, including sedimentation, phase separation, or undesired precipitation, during repeated thermal cycling. While nucleation agents can effectively reduce the degree of supercooling in most ISH systems at sufficiently high loading levels, in some cases, such as Glauber’s salt, the supercooling can remain as high as 10 °C even with as much as 10 wt % nucleation agent added.^44^ Adding large amounts of nucleation agent is ultimately detrimental to the heat storage capacity of the salogel as these inert particles neither store thermal energy nor provide shape stabilization.

**Figure 3:**
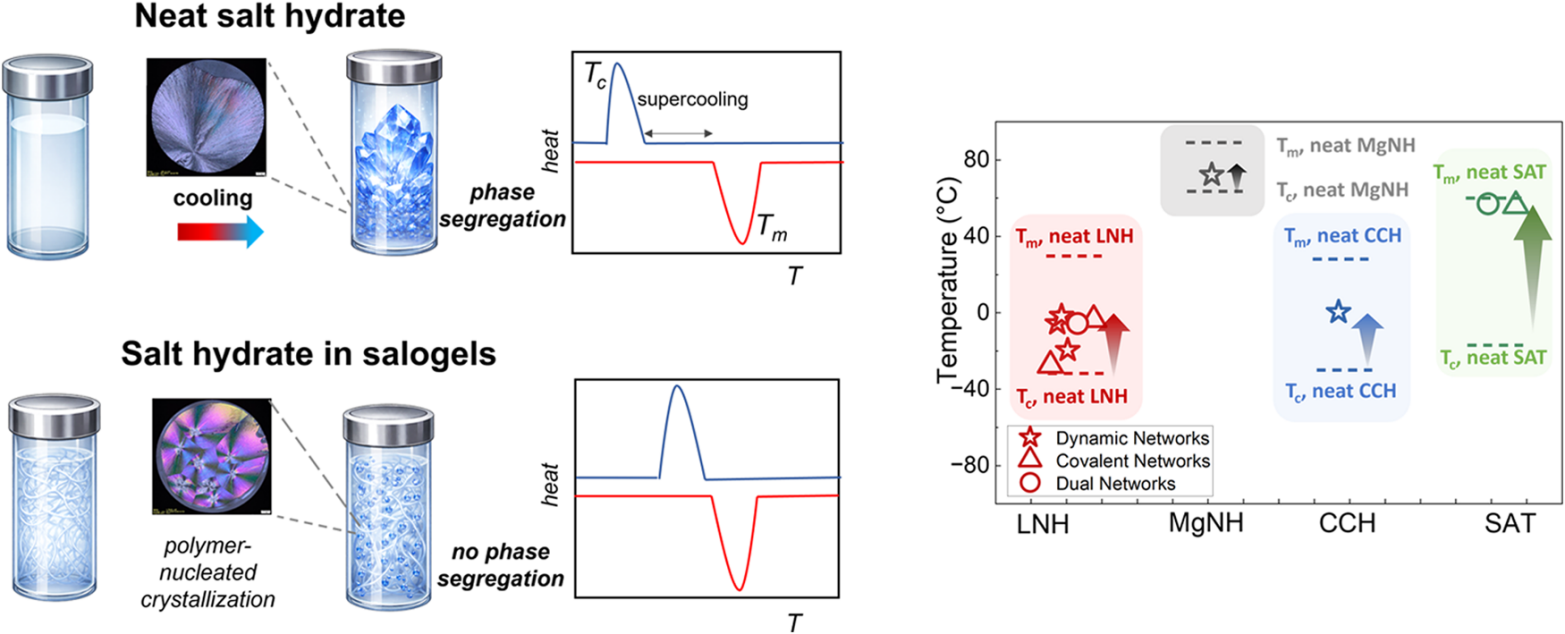
Schematic showing nucleation of neat ISH and ISH trapped in a polymer network (left). (Right) Plot showing the effect of the polymer network on the crystallization temperature of ISH PCMs in a salogel in the absence of a nucleation agent. The dotted lines indicate the melting and crystallization temperatures of the neat ISH. The data points indicate crystallization temperatures measured in the salogels. Data taken from refs 9,32–34,36,53,55,57,58 .

In contrast, polymer networks can potentially serve several purposes, including shape stabilization, heat of fusion retention, and supercooling suppression (schematics in Figure 3). The recent literature on salogels consistently shows that the polymer matrix often promoted the reduction of supercooling in a variety of salt hydrates for both dynamic and covalently crosslinked networks in the absence of nucleation agents.^9,33,34,36,53,55,57,58^ In the case of SAT, supercooling was almost completely eliminated in single^34^ and dual^36^ PAAm networks. Overall, the fundamental mechanism behind this reduction in supercooling in a gel remains poorly understood. We suggest, however, that the process can be similar to the interaction between biomolecules and inorganic ions during biomineralization yielding the biological organic–inorganic structures (e.g., bones, teeth, and shells)^111,112^ or between the polymers and inorganic precursors yielding synthetic organic–inorganic materials (e.g., perovskite single crystals^113^). In both cases, coordination of inorganic ions by polymer functional groups is seen as an important step for controlling the nucleation and growth of the inorganic phase. Future work in mitigating the supercooling in salt hydrates should move from nucleator selection alone to focusing on understanding the crystallization kinetics and nucleation onset in polymer salogels. One key unresolved issue is how polymer chemistry, crosslink density, and network architecture quantitatively influence the nucleation barrier and its sensitivity to external perturbations. Although polymer network parameters, such as polymer mesh size and polymer chemistry, have been demonstrated to affect nucleation kinetics, current understanding remains largely empirical.^114^ As a result, the field still lacks predictive relationships that quantitatively link the polymer structure to changes in the nucleation barrier height or triggering sensitivity. The design of future salogels can be guided by these studies so that apart from providing shape stabilization to the ISH, the polymer network should also work as a nucleation agent.

Apart from nucleation agents and confinement in polymer matrices, the use of external triggers such as electrical stimulation has emerged as a reproducible method for initiating nucleation in supercooled salt hydrate systems. For example, in SAT salogel systems based on cross-linked p­(AA-*co*-AAm) networks, application of an electric field induces localized crystallization in the vicinity of the electrode.^115^ Mechanistic studies showed that electric fields promoted ion migration and interfacial polarization, leading to local accumulation of ions and dehydration of salt hydrate clusters near the electrode, which reduced the nucleation barrier.^115^ In SAT-based latent heat storage devices, repeated electrical triggering produced consistent crystallization onset and localization, demonstrating the reversibility of the supercooled state under cyclic stimulation.^116^ In particular, studies using Ag electrodes with SAT crystals physically embedded within surface grooves of the electrode showed that nucleation preferentially occurred at the anode, indicating that local interfacial electrochemical conditions governed the crystallization onset.^116^ The mechanism was attributed to field-induced ion migration and dehydration of Ag^+^–acetate complexes near the electrode interface, which lowers the local kinetic barrier for nucleation.^116^

In addition to the electric field, ultrasound has been shown to initiate crystallization in supercooled salt hydrate systems through cavitation-induced pressure and temperature fluctuations.^117,118^ In contrast to nucleation in electrically triggered salt hydrates, ultrasonic stimulation typically results in widespread crystallization rather than localized control. For SAT, the application of ultrasonic waves generated localized nucleation events that rapidly propagated through the bulk once they were initiated.^118^ The mechanism was attributed to acoustic cavitation, where transient bubble collapse produces localized high-pressure regions that destabilize the metastable liquid and promote the formation of critical nuclei.^118^ However, the effectiveness of ultrasound to induce crystallization is strongly dependent on the sensitivity of a specific salt hydrate to transient pressure and temperature fluctuations.^118^ Mechanical perturbations such as shock, tapping, or agitation can also initiate nucleation in supercooled liquids.^119,120^ In supercooled SAT systems, transient mechanical disturbances were shown to destabilize metastable cluster populations and induce rapid crystallization.^119,120^ This behavior reflects the sensitivity of nucleation to transient stress fields, which can disrupt hydration structures and lower the kinetic barrier for nucleus formation.^119,120^ Taken together, supercooling mitigation strategies in ISH PCMs should progress from relying on the use of nucleation agents alone to designing salogel systems that can aid in the nucleation of salt hydrate crystals. However, fundamental research aimed at understanding polymer–ISH interactions that drive nucleation using experimental, computational, and ML tools is required. The development of novel salogel systems combined with external triggering, where such means can be employed, can be an effective means to eliminate supercooling.

## 3 Applications of Salogels in Thermal Management and Beyond

In this section, we highlight and discuss the various applications of polymer-stabilized inorganic salt hydrate gels or salogels (Figure 4). As mentioned in the Section 1, the appealing features of ISHs for thermal energy storage applications include their high volumetric latent heat, low cost, and suitable phase transition temperatures near ambient conditions.^5,6,121^ Moreover, the unwanted fluidity can be efficiently countered by entrapping ISHs with the polymer networks of the salogels.^9,31–52,55,57,70,81,92^ Taken together, these characteristics make ISH-containing salogels highly suitable for traditional thermal management applications such as in buildings, electronics, clothing, and waste heat recovery.^20,37,38,41,44,47,50,51,57,84,102,122^ Additionally, recent years have seen the emergence of newer applications of salogels in wearable devices, thermotherapy, energy conversion systems, and anti-icing and as supercapacitors and gel electrolytes in electronics.^36,43,45,52,57,69,84–86,92^ The use of ISHs in all these applications primarily requires shape stabilization and scalability among other attributes such as mechanical compliance, suppression of supercooling, and adhesion to various surfaces, all of which are afforded by salogels.^20,43,47,53,54,57^

**Figure 4:**
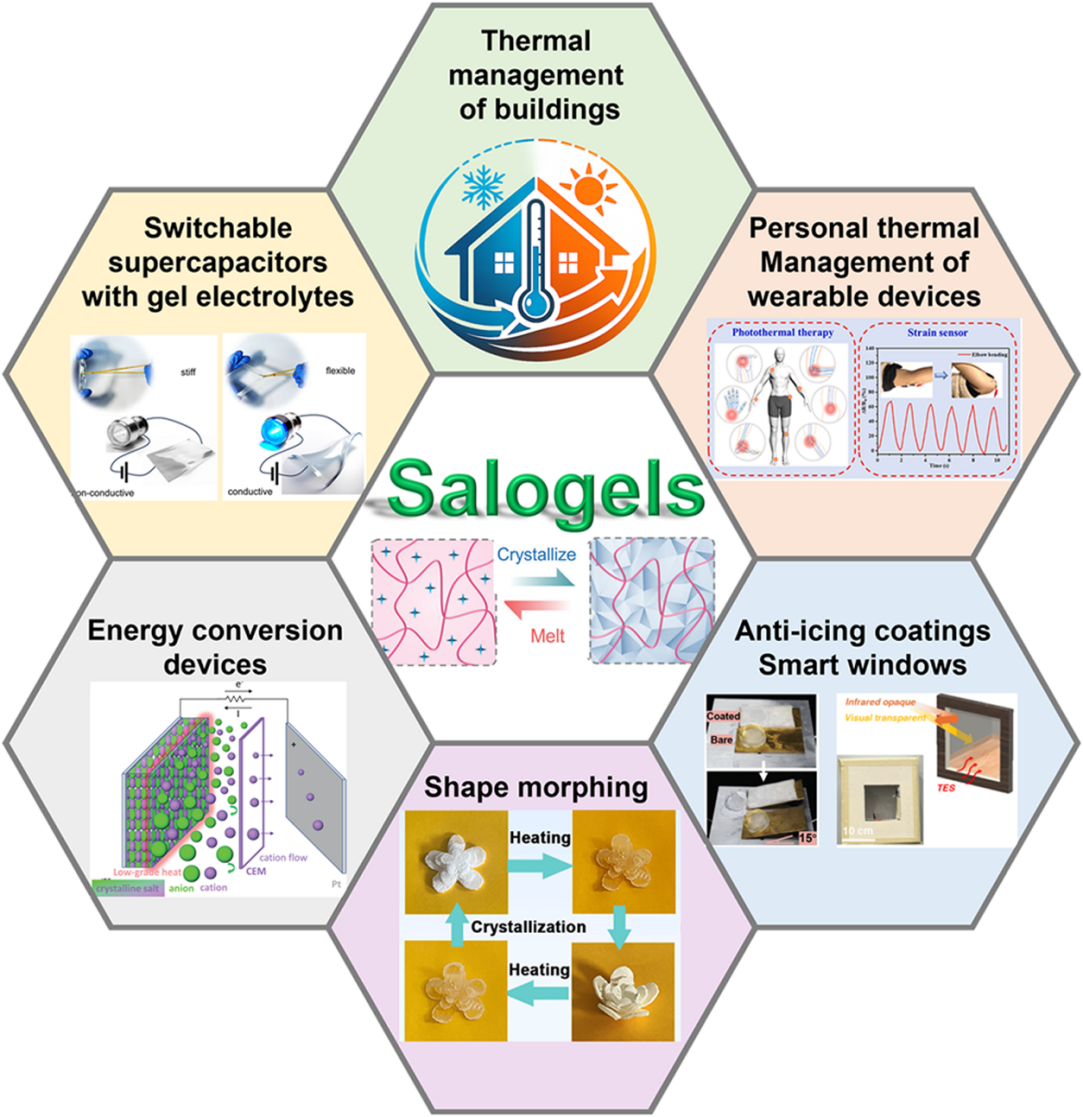
Applications of salogels. Images reproduced with permission from refs 44 (Copyright 2025 Elsevier),^46^ (Copyright 2023 Elsevier),^57^ (Copyright 2024 Wiley-VCH GmbH),^82^ (Copyright 2024 Wiley-VCH GmbH),^88^ (Copyright 2024 Elsevier),^126^ (Copyright 2023 Elsevier), and^128^ (Copyright 2021 Royal Society of Chemistry).

### 3.1 Thermal Management Applications in Buildings, Textiles, and Electronics

The latent heat stored in the materials during the ISH crystalline-to-melt phase transition can be used for building thermal management via passive and/or dynamic routes.^102^ In passive applications, the TES material is incorporated as part of the building structure such as in roofs or walls or windows, relying on the diurnal temperature changes for the thermal transitions to occur. In dynamic applications, the material is loaded into a TES module, such as air conditioning units and water heating systems, and the thermal transitions are controlled by heat transfer fluids or other means such as Joule heating. The effectiveness of the use of salt hydrate gels for building thermal management has been demonstrated using small model setups.^38,41,44,57^ Liu et al. demonstrated a floor heating system using a polymer gel containing SAT loaded into three parallel pipes fitted under the floor of a model house.^41^ Controlled crystallization of SAT using a crystal seed allowed heat release from one, two, or all three pipes as desired. Yin et al. demonstrated the use of a salt hydrate gel for the thermal management of buildings by applying the gel to the walls of a 3D-printed house as a coating.^57^ The temperatures inside the model house were maintained at a value ∼20 °C lower or higher during the melting or crystallization, respectively, compared to a control model house without the coating.

In addition to the TES capability, salt hydrate gels can provide the dual benefit of thermal management and optical adaptability in their application as smart windows.^44,57^ During the day, the polymer-entrapped salt hydrate melted and became transparent while cooling the indoor environment. During the night, the material’s crystallization released heat and provided privacy to the user due to the opaque state of the solid salogel. The simultaneous control of optical and TES properties, taken together with ease of scalability, good mechanical properties, and interfacial adhesion, presents significant advantages of the salogel-based approach compared to other smart window materials such as hydrogel and VO_2_ windows.^57^

Apart from thermal management of buildings, salogels are used in protective clothing^20,47^ due to their nonflammability—a property not achievable with organic PCMs. The shape-stable salogels, molded into a thin rectangular sheet and embedded in the lining of the protective clothing, were able to maintain a constant temperature, which was about 10–15 °C lower for 0.5–4 h in comparison to the case without the salogel protection.^20,47^

Another emerging application of salogels for thermal management is their use in electronic devices and batteries,^37,50,51,84,122^ where thermal runaways with temperatures rising to several hundred degrees can occur. The ability of inorganic salt hydrate PCMs to absorb large amounts of heat and their nonflammability make shape-stable salogels viable materials for this application compared to the traditional potting compounds (thermosetting resins) based on epoxies. Lu et al. showed that during a thermal runaway, the temperature of the battery pack with the PCM gel built from covalently crosslinked PAAS and sodium alginate was ∼200 °C lower than that of the commercial potting compound.^50^ Liao et al. demonstrated the use of salt hydrate gels for thermal management of a photovoltaic cell where the use of PCM maintained the temperature of the device at ∼80 °C lower than when there was no TES material present.^51^ In battery thermal management applications, salogels can help prevent thermal runaway reactions by making use of phase transitions beyond the traditional crystalline–melt phase change. These include thermochemical reactions of ISHs, which involve dehydration and hydration of the material. For example, the thermal runaway prevention of a battery was demonstrated for SAT- and CCH-based salogels.^122,123^ Therefore, the field of shape-stable ISH salogels is rapidly progressing from traditional building thermal management applications to cooling electronics and designing multifunctional smart windows. However, these examples demonstrate the capability of salogels to work as TES materials in a small-scale module under laboratory conditions and further work is required to demonstrate the scalability, reprocessability, and stability of salogel performance as a function of thermal cycling under real-world application conditions.

### 3.2 Personal Thermal Management and Wearable Devices

Another set of thermal management applications involves the use of salogels as wearable devices for personal thermal management.^36,43,45,46,52,57,69,84–86,92^ Personal thermal management applications include the use of PCMs to provide comfort to the human body through local temperature regulation and therapeutic applications such as thermotherapy or hot compresses. While the heat absorbed by salogel-entrapped ISHs provides cooling comfort, the heat released by the salogel can enable expansion of blood vessels and surrounding tissue, allowing increased blood flow and alleviation of pain in the joints.^46^ These applications are enabled by the flexibility and stretchability of the salogels that cannot be achieved by other PCM entrapment techniques, such as impregnation in rigid porous substrate matrices. Moreover, the use of polymers also allows adhesion of salogels to many types of substrate materials, enabling a broad range of applicability. In personal thermal management applications, flexible salogels can be applied to the human skin directly^57^ or after encasing within a plastic bag or textile.^32,43,45,84^

One approach to further enhance flexibility to salogels in their solid state is through encapsulation in a gel of organic PCM^92^ at a temperature when the ISH is molten. The following temperature-triggered ISH crystallization results in the release of the stored heat by the salogel. The local increase or decrease in temperature (10–20 °C) compared to the ambient temperature observed during the ISH phase transition confirms the feasibility of the approach.

In addition to personal thermal management, salogels can also be used as strain-sensing devices that can be attached to the human skin and can show high sensitivity to environmental stimuli and human body movements.^46,85,90^ However, these applications require enhancing the electrical conductivity of the salogels by adding conductive fillers such as MXene,^46^ graphene nanoplatelets,^85^ and polydopamine-coated nanoparticles.^90^ The flexibility, stretchability, and adhesion of the salogels to human skin along with enhanced electrical conductivity allow the conversion of mechanical deformation to electrical signals for real-time monitoring of physiological movements. The salogel-based sensors demonstrate high strains (>300%) and gauge factors (2.5–4.36), along with the stable strain sensing performance after multiple deformation and measurement cycles.^46,85,90^ These sensors respond to various movements of the human body such as movement of the hands, wrist, fingers, and elbows and even facial movements with performance comparable to or even superior to that of some hydrogels.^46,85,90^ The salogels containing conductive additives can be used for multiple purposes, including traditional TES applications, along with personal thermal management, and also for solar-to-thermal energy conversion as discussed in the following section. However, the heat of fusion retention of these salogels was less than 75%, highlighting the need for a rational salogel design strategy to improve TES performance while achieving the desired mechanical properties and performance characteristics for multifunctional applications.

### 3.3 Solar–Thermal Energy Conversion

Additives such as Mxene, graphene nanoplatelets, graphene oxide, polydopamine-coated nanoparticles, CuS, or carbon nanotubes (CNTs) included within salogels also enable solar-to-thermal energy conversion due to the photothermal effect.^34–36,46,48,79,85,90^ The high photothermal conversion efficiencies of 85–95% of conductive salogels^34–36,79^ make them promising for the use for solar–thermal energy conversion and photothermal therapy.^36,46,85^ In addition, the additive-improved (i.e., CNTs) electrical conductivity of the salogels can enable electrothermal energy conversion.^79^ Thus, conductive salogels can be used as both photothermal and electrothermal energy conversion devices. Future research can include the new emerging 2D nanomaterials, such as double-transition metal MXenes^124^ or graphdiyne,^125^ taking advantage of their high thermal and electric conductivity, as well as intrinsic porosity of the latter.

The thermal energy stored in a salogel can also be converted to electricity using thermoelectric devices based on the Seebeck effect.^40^ In this application, the thermal energy released during crystallization of the salogel to one side of a thermoelectric device creates a temperature difference between the hot and the cold sides and the electric current that is sufficient to light a LED bulb, charge a phone, or power humidity and temperature detectors for a few minutes.^40^ Therefore, salogels are not merely TES materials but can enable energy conversion and storage, making them truly multifunctional. However, current reported salogels achieved multifunctionality at the cost of TES performance (heat of fusion retention: 50–75%),^36,40,46,79,85^ highlighting the need for rational design of the polymer matrix to achieve balance of performance properties across applications. Moreover, the performance of salogels over multiple cycles in these applications remains unexplored and should become the focus of future studies.

### 3.4 Applications Based on Ionic Transport in Salt Hydrates

Recent studies demonstrate that ISH-based systems can simultaneously enable thermal energy storage and ion transport for electrical energy conversion and storage within a single material platform in the form of thermal-electrochemical^39,80,88,121,126,127^ and thermo-osmotic systems.^128^ Some representative examples include the development of phase change electrolytes based on SAT,^39,88,127^ sodium thiosulfate,^127^ SSD,^80^ and SSD–sodium thiosulfate mixtures.^121^ These electrolytes exhibit a high capacitance of ∼200 F g^–1^ and ionic conductivity exceeding 10 mS cm^–1^ in the liquid state of ISH, outperforming traditional materials such as polymer, hydrogel, and ceramic electrolytes,^39,121^ whereas the conductivity of salt hydrate-based electrolytes is reduced in the crystalline state; this reduction can be used for switchable supercapacitor applications.^39,80,88,126^ Stable electrochemical operation of ISH-based materials during repeated thermal cycling has been demonstrated, highlighting the robustness of ion transport under dynamic phase transitions. These systems demonstrate that ISHs can function as dual-purpose materials, where phase transitions not only store heat but also sustain electrochemical ion transport, enabling integrated thermal-electrical energy storage.

A direct coupling between thermal energy and ion transport is demonstrated in thermo-osmotic ionogel systems, where phase transitions actively generate ion gradients for energy conversion.^128^ In these systems, melting of polymer-confined LiNO_3_·3H_2_O generated a concentration gradient of mobile ions that governed selective ion diffusion through a cation exchange membrane. This system produced a significant electrical output, with a single device achieving an open-circuit voltage of 0.52 V, a thermopower of 26 mV K^–1^, and a peak power density of 0.4 W m^–2^ under a 90 °C heat source. Notably, the system reached a peak heat-to-electricity conversion efficiency of 11.17%, which was significantly higher than what has been achieved with conventional low-grade heat recovery technologies, including thermoelectric systems (<4.1%), pyroelectric systems (<3.8%), and thermo-electrochemical systems (<5.7%). This enhanced performance arises from osmotic-driven ion transport, which is more efficient than temperature gradient-driven diffusion and enables effective conversion of thermal energy into ionic and electrical outputs. These results highlight that ISHs can be engineered not only to store thermal energy but also to actively convert heat into electricity via ion transport mechanisms, representing a significant advancement over traditional energy conversion approaches. However, the TES performance of these systems was relatively low, achieving <50% retention of heat of fusion.^88,127^ Future work should focus on achieving a balance of electrical and thermal properties along with evaluation of thermal and electrical performance over multiple cycles to enable salogels to become truly multifunctional materials.

### 3.5 Other Applications

One interesting application of salogels synergistically uses the PCM properties of salogels along with the hygroscopicity of salt hydrates to provide thermal regulation and moisture control in food packaging. In this application, the dual function of the salogels supported the retention of the freshness of fruits during transportation.^42,89^

Several other recent applications go beyond thermal energy storage or the energy conversion potential of salogels. In one such application, Yin et al. demonstrated frost resistance and anti-icing capabilities of the salogel coatings.^57^ The anti-icing behavior was enabled by the melting point suppression of water and heat released during crystallization of the ISH, which prevented the formation of ice.^57^ Because of the presence of a network of polar polymers, the salogels strongly adhered to various substrates, enabling their wide usage for this application.

Other applications such as smart windows are based on changes in transparency^44,57^ and a 3–4 orders of magnitude increase in stiffness occurring during crystallization of polymer-trapped salt hydrates.^39,40,67,69,82,88^ The transition from a soft to a hard material during phase transition has also been exploited for controlling materials’ shape morphing,^67,69,82^ where the hard crystalline state of the salogel’s ISH supported shape fixity and the transition to the soft gel state was used for shape recovery. Unlike the shape memory behavior of polymers which require external temperature changes, the supercooling phenomenon allows existence of the soft, supercooled ISH or hard, crystalline state at the same temperature,^82^ so that external triggering of crystallization (e.g., by crystal seeds) can be used to initiate shape morphing. However, the TES performance of these salogels was not reported^67,69,82^ and should be addressed in the future to enable the development of advanced shape-morphing TES materials.

## 4 Conclusions and Outlook

Due to the high salt content, high volumetric latent heat, low cost, and non-flammability, salt hydrate-based materials offer a compelling platform for many applications in the energy sector and beyond. Polymer–salt hydrate hybrid materials, termed as salogels, have emerged as versatile shape-stabilized, scalable materials that can preserve the high thermal energy density and potentially reduce supercooling in ISHs. These applications largely include energy-centered uses for thermal energy storage. Recent advances in crosslinking approaches in polymer salogels using dynamic bonds, such as boronate ester and Diels–Alder chemistries, have allowed the development of reprocessable and self-healable salogels with tunable gelation and mechanical properties. At the same time, emerging applications highlight opportunities for the multifunctionality of the salogels making use of their mechanical and optical properties, ionic conductivity, adhesion, photothermal, and electrothermal energy conversion to expand the field beyond traditional thermal management applications.

Despite this progress, several key challenges remain to ensure that salogels can be employed in real-world applications that offer rich opportunities for future research and broad applications beyond TES (Figure 5). Achieving a combination of robust mechanical properties along with high TES capacity and reprocessability is highly desired. Moreover, because current studies often sacrifice thermal performance while achieving multifunctionality, it is important to develop multifaceted and balanced performance characteristics for these materials.

**Figure 5:**
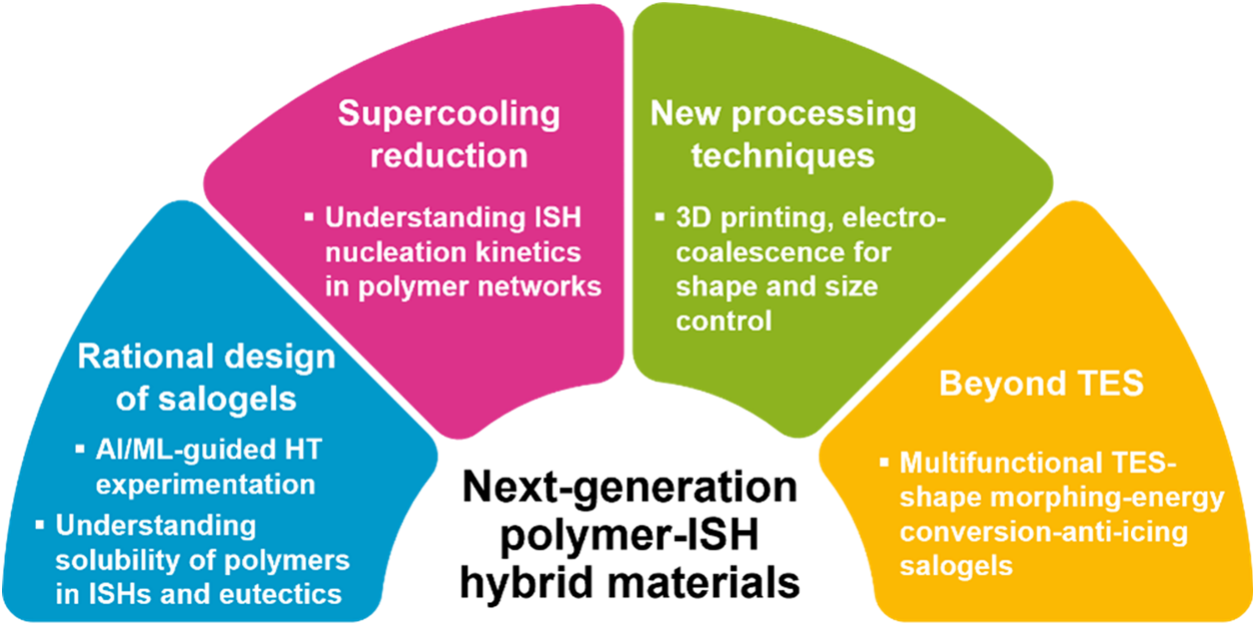
Outlook for the future directions in the development of next-generation salogel materials.

Future developments of salogels should be rooted in the fundamental understanding of polymer–ISH interactions using experimental and computational techniques along with using artificial intelligence (AI) and machine learning (ML) tools. To date, salogel design has employed a trial-and-error approach using a limited set of polymers and salt hydrates. However, the solubility of many polymers in a variety of ISHs remains unexplored. Following the success of ML algorithms in predicting polymer solubility in organic solvents,^129–132^ future research can expand the applications of these techniques to predict polymer solubility and assembly in the complex multicomponent environment of inorganic salt hydrate solvents. The development of ML-guided correlations between the polymer chemical structure and solubility in salt hydrates can enable the synthesis of polymers with targeted chemical composition and molecular architecture for controlling thermomechanical and ionic conductivity characteristics of the next generation of salogels.

So far, salogel research has been focused on individual ISHs. However, eutectic ISHs, which allow tunability of phase transition temperatures based on application and mitigate phase segregation and supercooling,^4,5,133^ remain unexplored. Eutectic ISHs can enable the design of new salogel systems with reduced supercooling and tunable phase transition temperature for different applications. However, the effect of multiple ion types on polymer solvation and gelation in eutectic ISHs needs to be studied to enable the rational design of salogel systems. Therefore, future work should focus on integrating high-throughput experimentation with ML, offering a transformative approach for salogel design, enabling predictive mapping of polymer–ion–water interactions in single and eutectic ISHs to gelation, thermal performance, and mechanical properties.^134,135^

The challenge of supercooling in salogels presents an opportunity for future research. Fundamental work geared toward understanding polymer–ISH interactions from the perspective of nucleation and crystallization is required so that next-generation salogels are not only shape-stable but also self-sufficient in terms of supercooling reduction. Researchers can draw insights from natural and synthetic inorganic materials to approach this challenge.

The use of polymer matrices that not only achieve shape stability but also provide phase change capability can comprise another promising future direction. These future advanced polymer matrices based on semicrystalline polymers, such as PEG, can add to the latent heat stored by the material, thereby increasing the TES capacity even when used in large amounts to control mechanical properties.

While current salogels are created by using traditional molding techniques, new processing techniques to create form factors for polymer–ISH materials that could enhance heat transfer are required. To this end, the use of advanced processing techniques, including 3D printing, is desired to enable the fabrication of complex, high-surface area geometries that reduce thermal resistance within PCM devices.^102^ Coupling these design capabilities with mechanical and thermal property enhancements of salogels could substantially broaden their applications beyond thermal energy storage to flexible devices and multifunctional energy storage systems. Note that 3D printing has been demonstrated only for organic PCMs and remains unexplored for ISH PCMs, being hindered by the sensitivity of the material to environmental conditions (hygroscopicity and dehydration).^102^ One approach to overcoming the hygroscopicity problem has been encapsulating the salogel in plastic bags^32,43,45,84^ or constructing sandwich structures where the ISH PCM gel is encapsulated within an organic PCM gel.^92^ This approach can not only solve the hygroscopicity problem but also enable 3D-printed organic–inorganic hybrid materials that utilize the TES capabilities of both organic and ISH PCMs. Another promising new direction for processing salogels is achieving microscale control of dimensions in TES materials by creating fibers^122,136^ and microscale shape-stable gel particles.^137^ Fibers, due to their anisotropy, can provide fast heat transfer pathways in the in-plane direction, achieving excellent heat transfer properties.^122^

Finally, it is important to integrate and optimize the innovations in materials design and processing with the multifunctionality of future salogel materials. Despite the recent emergence of new applications in electrochemical conversion, supercapacitors, and solid electrolyte membranes, the large body of applications for polymer–ISH hybrid materials is exclusively centered on thermal energy storage. New applications that leverage the multifunctionality of salogels as materials which support latent heat storage, ionic transport, and shape morphing capability are of great potential and should be more actively pursued.

## Abbreviations

ISH: inorganic salt hydrate
PCM: phase change material
TES: thermal energy storage
PVA: poly­(vinyl alcohol)
PAAm: poly­(acrylamide)
LNH: lithium nitrate trihydrate
CCH: calcium chloride hexahydrate
MgNH: magnesium nitrate hexahydrate
DHPD: disodium hydrogen phosphate decahydrate
SSD: sodium sulfate decahydrate
SAT: sodium acetate trihydrate
ATR-FTIR: attenuated total reflectance-fourier transform infrared spectroscopy
NMR: nuclear magnetic resonance
DFT: density functional theory
p­(AAm-*co*-HEMA): poly­(acrylamide-*co*-hydroxyethyl methacrylate)
PAA: poly­(acrylic acid)
PAAS: sodium salt of poly­(acrylic acid)
